# Leading causes of death in Vietnamese Americans: An ecological study based on national death records from 2005–2020

**DOI:** 10.1371/journal.pone.0303195

**Published:** 2024-05-24

**Authors:** Khoa Tran, HyeYuong Shon, Jonathan Phan, Tina Cheng, Gloria S. Kim, Armaan Jamal, Malathi Srinivasan, Latha P. Palaniappan, Linda Nguyen, Robert J. Huang

**Affiliations:** 1 Department of Humanities, New York University, Abu Dhabi, United Arab Emirates; 2 Department of Psychology, University of California, Berkeley, CA, United States of America; 3 Department of Bioengineering, University of California, Los Angeles, CA, United States of America; 4 Center for Asian Health Research and Education, Stanford University School of Medicine, Stanford, CA, United States of America; 5 Division of Cardiovascular Medicine, Stanford University School of Medicine, Stanford, CA, United States of America; 6 Department of Medicine, Johns Hopkins University School of Medicine, Baltimore, Maryland, United States of America; 7 Division of Primary Care and Population Health, Stanford University School of Medicine, Stanford, CA, United States of America; 8 Division of Gastroenterology and Hepatology, Stanford University School of Medicine, Stanford, CA, United States of America; Oklahoma State University, UNITED STATES

## Abstract

**Background:**

Disaggregated data is a cornerstone of precision health. Vietnamese Americans (**VietAms**) are the fourth-largest Asian subgroup in the United States (**US**), and demonstrate a unique burden of disease and mortality. However, most prior studies have aggregated VietAms under the broader Asian American category for analytic purposes. This study examined the leading causes of death among VietAms compared to aggregated Asian Americans and non-Hispanic Whites (**NHWs**) during the period 2005–2020.

**Methods:**

Decedent data, including underlying cause of death, were obtained from the National Center for Health Statistics national mortality file from 2005 to 2020. Population denominator estimates were obtained from the American Community Survey one-year population estimates. Outcome measures included proportional mortality, age-adjusted mortality rates per 100,000 (**AMR**), and annual percent change (**APC**) in mortality over time. Data were stratified by sex and nativity status. Due to large differences in age structure, we report native- and foreign-born VietAms separately.

**Findings:**

We identified 74,524 VietAm decedents over the study period (71,305 foreign-born, 3,219 native-born). Among foreign-born VietAms, the three leading causes of death were cancer (26.6%), heart disease (18.0%), and cerebrovascular disease (9.0%). Among native-born VietAms the three leading causes were accidents (19.0%), self-harm (12.0%), and cancer (10.4%). For every leading cause of death, VietAms exhibited lower mortality compared to both aggregated Asians and NHWs. Over the course of the study period, VietAms witnessed an increase in mortality in every leading cause. This effect was mostly driven by foreign-born, male VietAms.

**Conclusions and relevance:**

While VietAms have lower overall mortality from leading causes of death compared to aggregated Asians and NHWs, these advantages have eroded markedly between 2005 and 2020. These data emphasize the importance of racial disaggregation in the reporting of public health measures.

## Introduction

The end of the Vietnam War in 1975 sparked a mass migration of many Vietnamese nationals to the United States (**US**). Five decades later, the Vietnamese American (**VietAm**) diaspora now comprises the fourth-largest Asian subgroup within the US, with a population of 2.2 million [1, 2]. Despite constituting a significant portion of the Asian American population, research focused on VietAm health and mortality is scarce. Most prior literature aggregated VietAm under the broader Asian American racial category, and therefore analyzed VietAm health data along with other Asian American subgroups.

While there have been health research literature derived from Vietnam, the unique story of immigration and the effects of dietary and lifestyle acculturation on the VietAm population make this a distinctive group that requires study [3, 4]. Understanding health comprehensively necessitates considering social determinants [5]. Vietnamese nationals and VietAms have different lifestyles, health behaviors, and socioeconomic statuses. For example, Vietnam is a developing country with a tropical climate, which faces a different profile of infectious tropical diseases [6]. In addition, longitudinal studies focused on the health trends of Asian immigrants have found significant correlations between acculturation and higher risks of excess body mass, diabetes mellitus, and poor cardiovascular health [7]. Immigrants are at greater risk for cardiometabolic disease compared to inhabitants of their country of origin [8, 9]. Therefore, there is a need to examine the leading causes of mortality among VietAms, rather than relying on studies conducted in Vietnam.

Within the VietAm population, the socioeconomic status of native-born and foreign-born individuals significantly differ. Native-born VietAms are younger, better-educated, and richer than foreign-born VietAms [2, 10]. These differences illustrate how native-born and foreign-born VietAms occupy different socioeconomic strata, which in combination with other determinants, impact health. For these reasons, this study aimed to characterize the leading causes of death among VietAms and analyze the trends in both native-born and foreign-born VietAms to gain a better understanding of their health status and needs. Due to the unique immigration history experienced by the VietAm population, we sought to compare patterns of mortality between VietAms and aggregated Asian Americans. We also sought to compare VietAm mortality with the mortality patterns of non-Hispanic White (**NHW**) Americans.

## Methods

### Study data and research ethics

This study was drawn from public and deidentified data sources, and did not constitute human subjects research (Stanford Institutional Review Board determination number 53429). The process for data collection, data cleaning and data analysis is summarized as a flowchart in **S1 Fig**. The National Center for Health Statistics US national mortality data sets contain comprehensive data on decedents derived from standard US death certificates, and are available for research use [11]. Beginning in 2003, the standard US death certificate began to provide detailed Asian racial groupings [12]. In most instances, the funeral director is responsible for collecting personal and demographic data on the decedent, either through inquiry of family members or through observation. Variables provided in the death certificates include the decedent’s race, sex, age, nativity status (based on location of birth), and identified primary cause of death. Mortality data in the US is overwhelmingly complete with low missing information on these variables. Using disaggregated racial codes, we identified all VietAm, aggregated Asian American, and NHW decedents over the study period 2005 to 2020. For the purposes of this study, the “aggregated” Asian American grouping represented the six largest Asian subgroups (Chinese Japanese, Filipino, Indian, Korean Vietnamese) which collectively represent >85% of the US Asian American population.

Using the International Classification of Diseases, 10^th^ revision (**ICD-10**) codes, the leading causes of both populations were characterized and grouped into twelve broader disease categories: heart diseases (I00-I09, I11, I13, I20-I51); malignant neoplasms (C00-C97); diabetes mellitus (E10-E14); cerebrovascular diseases (I60-I69); influenza/pneumonia (J09-J18); Alzheimer’s disease (G30); chronic liver diseases (K70-K76); nephritis, nephrotic syndrome, and nephrosis (N00-N07, N17-N19, N25-N27); chronic lower respiratory diseases (J40-J47); intentional self-harm (U03, X60-X84, Y87.0); accidents (V01-X59, Y85-Y86); deaths due to all other causes were classified as other [13, 14].

Total population counts (the denominator) were estimated using the American Community Survey 1-year population estimate from 2005 to 2020 available from the US Census Bureau. This data was accessed using the IPUMS USA database (University of Minnesota, Minneapolis, MN) [15]. The 1-year estimate provides estimates of the US population by disaggregated race, sex, age, and nativity status. The estimates also provide standard error estimates for each population strata based on sampling weights. In this survey, race is self-reported and we excluded mixed-race individuals.

Because the VietAm population has grown rapidly due to immigration over the last 50 years, the age distribution of native-born and foreign-born VietAms are significantly different (**S2 Fig**). To observe and account for these differences, we stratified all groups by age (0–19, 20–39, 40–59, and ≥60) and performed age-adjusted analyses. To perform the age-adjusted analyses, we first calculated the observed rates within each demographic bin are multiplied by the proportional size of that bin in a standard population. In this case, we utilized the year 2000 US Census population as the standard population.

### Statistical analysis

Using the mortality data and the population counts, we calculated the age-adjusted mortality rates per 100,000 person-years (**AMR**) with 95% confidence intervals (**CI**) for primary causes of death. We also calculated proportional mortality for each group and strata, defined as the number of deaths due to a given cause divided by total deaths. To evaluate secular trends, we compared the trendlines (2005–2020) of the leading causes of death within each group and calculated annual percent change (**APC**) in each leading cause of death. Analyses were stratified by sex and nativity status. All computational analyses and visualizations were completed using R statistical programming software Version 4.2.1 on RStudio and Microsoft Excel.

## Results

### Characteristics of populations and decedents

**Table 1** depicts the number of decedents and population person-years of observation by sex, age, and race. A total of 71,305 foreign-born VietAm, 3,219 native-born VietAm, 500,319 aggregated Asian, and 33,231,057 NHW decedents were identified from 2005 to 2020, respectively. Among foreign-born VietAms, 57% of decedents were male and 43% were female. Among native-born VietAms, 63% of decedents were male and 37% were female. Foreign-born VietAm decedents were predominantly older, with nearly 78% of decedents ≥60 years of age. By contrast, nearly half of native-born VietAm decedents were <20 years of age. Among both Asian Americans and NHWs, the large majority of decedents were older: 80% of Asian decedents and 84% of NHW decedents were ≥60, respectively.

**Table 1:** Demographic characteristics of the cohort.

Variable	Decedents	Population (person-years of observation)	Variable	Decedents	Population (person-years of observation)
	No. (%)	No. (%)		No. (%)	No. (%)
**Vietnamese (foreign-born)**			**Aggregated Asian Americans**		
Sex			Sex		
Male	40,624 (57.0)	8,409,890 (46.9)	Male	262,275 (52.4)	100,274,083 (47.3)
Female	30,681 (43.0)	9,521,065 (53.1)	Female	238,044 (47.6)	111,664,610 (52.7)
Age			Age		
0–19	188 (0.2)	968,723 (5.4)	0–19	10,357 (2.1)	43,809,008 (20.7)
20–39	2,343 (3.3)	5,097,191 (28.4)	20–39	19,718 (3.9)	67,328,849 (31.8)
40–59	13,313 (18.7)	7,678,390 (42.8)	40–59	69,657 (13.9)	62,240,682 (29.4)
≥60	55,461 (77.8)	4,186,651 (23.4)	≥60	400,587 (80.1)	38,560,154 (18.2)
**Vietnamese (native-born)**			**Non-Hispanic White**		
Sex			Sex		
Male	2,028 (63.0)	4,428,582 (51.6)	Male	16,577,356 (49.9)	1,551,964,725 (49.2)
Female	1,191 (37.0)	4,157,544 (48.4)	Female	16,653,701 (50.1)	1,601,205,592 (50.8)
Age			Age		
0–19	1,605 (49.9)	5,230,303 (60.9)	0–19	350,063 (1.1)	664,000,736 (21.1)
20–39	1,243 (38.6)	2,757,990 (32.1)	20–39	973,526 (2.9)	779,887,419 (24.7)
40–59	125 (3.9)	422,342 (4.9)	40–59	4,108,747 (12.4)	893,827,269 (28.3)
≥60	246 (7.6)	175,491 (2.0)	≥60	27,798,721 (83.7)	815,454,893 (25.9)

**Table 1:** Characteristics of Vietnamese American (foreign- and native-born), aggregated Asian Americans, and Non-Hispanic White population and decedents (2005–2020)

### Proportional mortality and age-adjusted mortality rates among Vietnamese Americans

Based on proportional mortality (**Table 2**), the three leading causes of death for VietAms were cancer (28.7%), heart disease (17.4%), and cerebrovascular disease (8.7%). When stratified by nativity status, the three leading causes of death among foreign-born VietAms were cancer (26.6%), heart disease (18.0%) and cerebrovascular disease (9.0%). By contrast, the three leading causes among native-born VietAms were accidents (19.0%), self-harm (12.0%), and cancer (10.4%). The three leading causes of death for aggregated Asian Americans were cancer (26.1%), heart disease (22.2%), and cerebrovascular disease (7.6%). Foreign-born VietAms were quite similar to aggregated Asians with regards to leading causes, and shared all ten leading causes (including the top five leading causes in the same order). For NHWs, the three leading causes were heart disease (23.7%), cancer (21.8%), and chronic lower respiratory diseases (6.0%).

**Table 2:** Leading causes of death by race (% of total deaths), in ranked order.

Vietnamese American (Overall)	Vietnamese American (foreign-born)	Vietnamese American (native-born)	Aggregated Asian Americans	Non-Hispanic White
Cancer (28.7%)	Cancer (26.6%)	Accidents (19.0%)	Cancer (26.1%)	Heart disease (23.7%)
Heart disease (17.4%)	Heart disease (18.0%)	Self-harm (12.0%)	Heart disease (22.2%)	Cancer (21.8%)
Cerebrovascular disease (8.7%)	Cerebrovascular disease (9.0%)	Cancer (10.4%)	Cerebrovascular disease (7.6%)	Chronic lower respiratory diseases (6.0%)
Accidents (4.7%)	Accidents (4.1%)	Heart disease (5.9%)	Accidents (4.0%)	Accident (5.1%)
Diabetes (3.7%)	Diabetes (3.8%)	Cerebrovascular disease (2.2%)	Diabetes (3.8%)	Cerebrovascular Disease (5.0%)
Chronic lower respiratory diseases (3.1%)	Chronic lower respiratory diseases (3.1%)	Chronic lower respiratory diseases (1.1%)	Influenza & Pneumonia (3.2%)	Alzheimer’s disease (3.9%)
Influenza & Pneumonia (2.5%)	Influenza & Pneumonia (2.6%)	Influenza & Pneumonia (1.1%)	Alzheimer’s disease (3.1%)	Diabetes (2.5%)
Alzheimer’s disease (2.4%)	Alzheimer’s disease (2.6%)	Diabetes (1.0%)	Chronic lower respiratory diseases (2.9%)	Influenza & Pneumonia (2.1%)
Self-harm (2.0%)	Self-harm (1.5%)	Kidney disease (0.7%)	Kidney disease (1.9%)	Chronic liver diseases (1.7%)
Kidney disease (1.9%)	Kidney disease (1.9%)	Alzheimer’s disease (0.3%)	Self-harm (1.2%)	Kidney disease (1.6%)

**Table 2:** Leading causes of death by proportion of total deaths among Vietnamese Americans (stratified by nativity), aggregated Asian Americans, and Non-Hispanic Whites from 2005 to 2020, in ranked order

The AMRs (reported per 100,000 person-years of observation), both overall and by cause, for each group are depicted in **Table 3**. VietAms had lower overall mortality (male mortality 206, 95% CI 189–241; female mortality 137, 95% CI 125–157) than aggregated Asians (male mortality 415, 95% CI 400–432; female mortality 322, 95% CI 312–335) and NHWs (male mortality 870, 85% CI 860–881; female mortality 715, 95% CI 707–724). VietAms also had lower mortality across disease categories, including lower mortality from cancer, heart disease, cerebrovascular disease, accidents, and diabetes. AMRs by nativity status are also depicted. While significant differences in AMRs exist between foreign- and native-born groups, this is likely driven in large part by fundamental differences in age structure. Among both foreign- and native-born VietAms, males exhibit higher mortality than females.

**Table 3:** Age-adjusted mortality rate per 100,000 person-years (95% CI).

Cause	Vietnamese Americans		Vietnamese (foreign-born)		Vietnamese (native-born)		Aggregated Asian Americans		Non- Hispanic White	
	Male	Female	Male	Female	Male	Female	Male	Female	Male	Female
Overall	206.4 (189.2–240.9)	136.6 (125.3–156.8)	437.5 (413.9–464.3)	290.9 (275.3–308.6)	51.5 (47.3–57.4)	27.8 (26.2–29.7)	415.4 (400.3–432.0)	322.8 (311.6–334.9)	870.4 (859.5–881.8)	715.2 (707.3–723.5)
Cancer	62.0 (56.3–75.5)	35.1 (32.3–39.6)	103.5 (97.8–109.9	59.7 (56.6–63.3)	20.3 (14.7–40.8)	10.4 (8.0–16.0)	100.4 (96.9–104.4)	76.1 (73.7–78.9)	199.6 (197.1–202.2)	156.2 (154.5–158.1)
Heart disease	36.4 (33.0–44.0)	24.3 (22.0–28.5)	59.7 (56.5–63.4)	39.6 (37.4–42.1)	12.8 (9.5–24.5)	9.0 (6.6–15.0)	103.2 (99.3–107.5)	69.6 (67.1–73.3)	223.3 (220.5–226.3)	168.5 (166.6–170.4)
Cerebrovascular disease	15.4 (14.1–18.6)	13.5 (12.3–15.9)	26.2 (24.8–27.9)	23.2 (21.9–24.6)	4.6 (3.3–9.2)	3.7 (2.7–7.2)	27.1 (26.1–28.2)	28.0 (27.0–29.1)	36.0 (35.5–36.5)	43.1 (42.6–43.6)
Accidents	14.2 (13.1–15.8)	5.7 (5.2–6.3)	18.7 (17.5–20.0)	8.0 (7.5–8.6)	9.8 (8.6–11.5)	3.3 (2.9–3.9)	19.6 (19.0–20.4)	10.5 (10.1–10.9)	66.1 (65.1–67.1)	32.8 (32.4–33.2)
Diabetes	5.8 (5.4–6.2)	5.4 (5.2–6.0)	10.7 (10.1–11.3)	9.4 (8.9–10.0)	0.8 (0.7–1.0)	1.4 (1.1–2.0)	15.7 (15.1–16.3)	10.7 (10.4–11.1)	23.0 (22.7–23.2)	16.0 (15.8–16.2)
Chronic lower respiratory disease	7.7 (7.1–8.7)	2.9 (2.6–3.5)	13.1 (12.3–13.9)	4.8 (4.6–5.1)	2.4 (1.8–3.4)	1.0 (0.7–1.8)	13.8 (13.3–14.4)	7.3 (7.0–7.5)	40.9 (40.4–41.3)	38.1 (38.8–38.5)
Self-harm	6.7 (6.2–7.2)	2.3 (2.2–2.6)	8.5 (7.9–9.2)	2.4 (2.3–2.6)	4.8 (4.5–5.3)	2.2 (2.0–2.6)	8.3 (8.1–8.6)	3.3 (3.2–3.4)	27.1 (26.7–27.6)	7.9 (7.8–8.1)
Influenza/ Pneumonia	4.5 (4.2–4.8)	3.5 (3.2–4.2)	8.4 (7.9–8.9)	6.0 (5.7–6.4)	0.6 (0.5–0.7)	1.1 (0.8–1.9)	14.8 (14.2–15.5)	11.0 (10.6–11.5)	18.0 (17.7–18.2)	16.1 (15.9–16.3)
Alzheimer	3.1 (2.8–3.7)	4.7 (4.3–5.2)	5.1 (4.9–5.5)	8.0 (7.6–8.5)	1.0 (0.7–1.9)	1.4 (1.1–1.9)	9.6 (9.2–10.0)	17.1 (16.4–17.8)	19.2 (19.0–19.4)	36.4 (36.1–36.8)
Kidney disease	3.0 (2.9–3.3)	2.7 (2.5–3.0)	5.6 (5.3–6.0)	4.6 (4.4–4.9)	0.5 (0.4–0.6	0.8 (0.7–1.2)	8.1 (7.8–8.5)	5.8 (5.6–6.0)	14.0 (13.8–14.2)	10.6 (10.5–10.7)
Chronic Liver disease	3.9 (3.6–4.5)	1.8 (1.7–2.1)	6.4 (6.1–6.8)	2.9 (2.7–3.1)	1.4 (1.0–2.1)	0.7 (0.6–1.0)	5.0 (4.9–5.2)	3.1 (3.0–3.2)	18.6 (18.4–18.8)	9.9 (9.8–10.0)

**Table 3:** Age-adjusted mortality rate of Vietnamese Americans (stratified by nativity status), Asian Americans, and non-Hispanic Whites from 2005 to 2020.

### Secular trends among Vietnamese Americans

**Fig 1:**
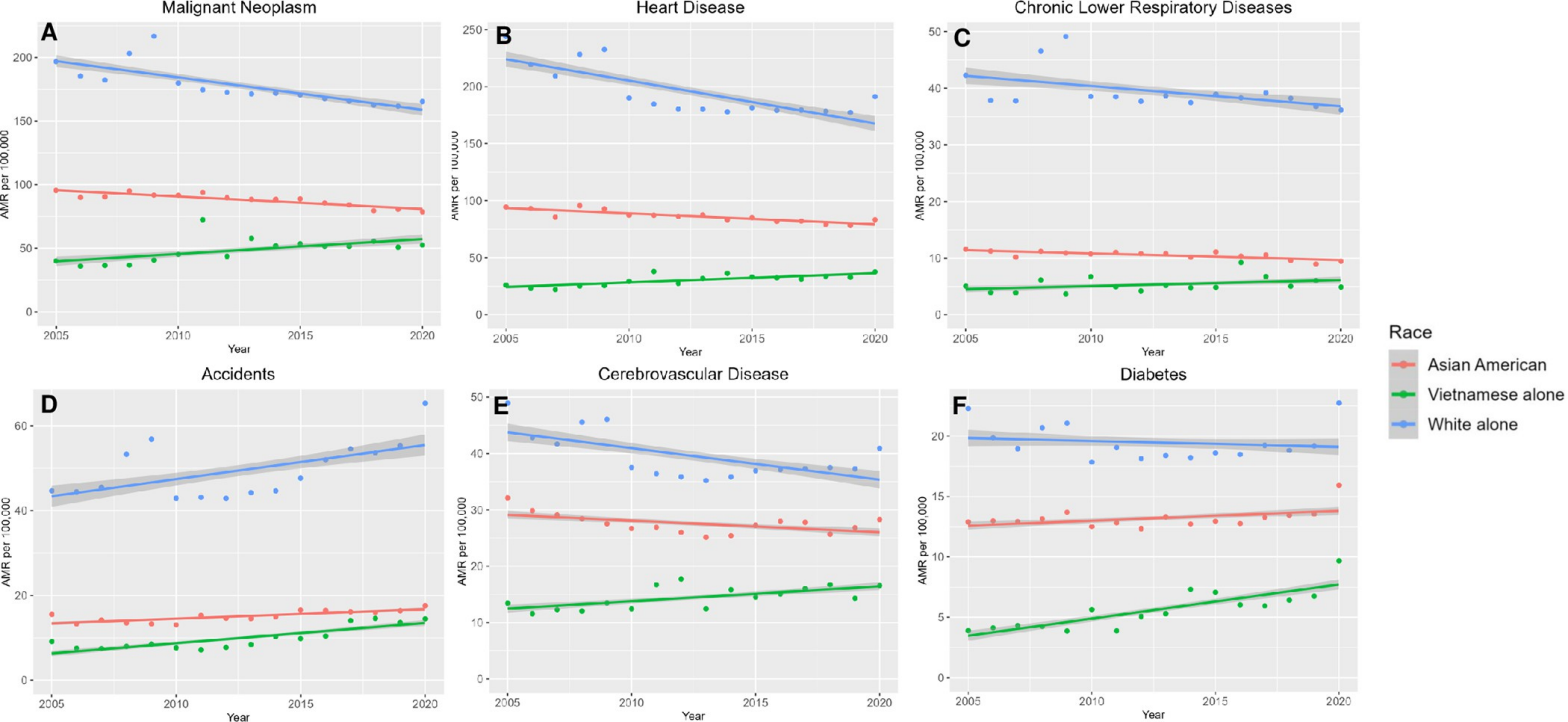
Age-standardized mortality rates from (A) cancer, (B) heart diseases, (C) chronic lower respiratory tract diseases, (D) accidents, (E) cerebrovascular diseases, and (F) diabetes among Vietnamese Americans, aggregated Asian Americans, and non-Hispanic Whites, 2005–2020.

Annual mortality across the study period (2005–2020) among VietAms, aggregated Asians, and NHWs for six leading causes (cancer, heart disease, chronic lower respiratory disease, accidents, cerebrovascular disease, and diabetes,) are plotted in **Fig 1** (corresponding data in **S1 Table**). Across these leading causes, NHW mortality decreased for most chronic diseases (cancer, heart disease, chronic lower respiratory disease, cerebrovascular disease; APC for all p<0.001), while mortality from accidents increased (APC 1.65%, p<0.001). By contrast, VietAms mortality increased over time for all six causes (APC p<0.01 for all). Aggregated Asian Americans demonstrated a mixed pattern. While mortality decreased for some diseases (cancer, heart disease, chronic lower respiratory disease; p<0.001), they increased for others (accidents, cerebrovascular disease, diabetes; p<0.05).

**Fig 2:**
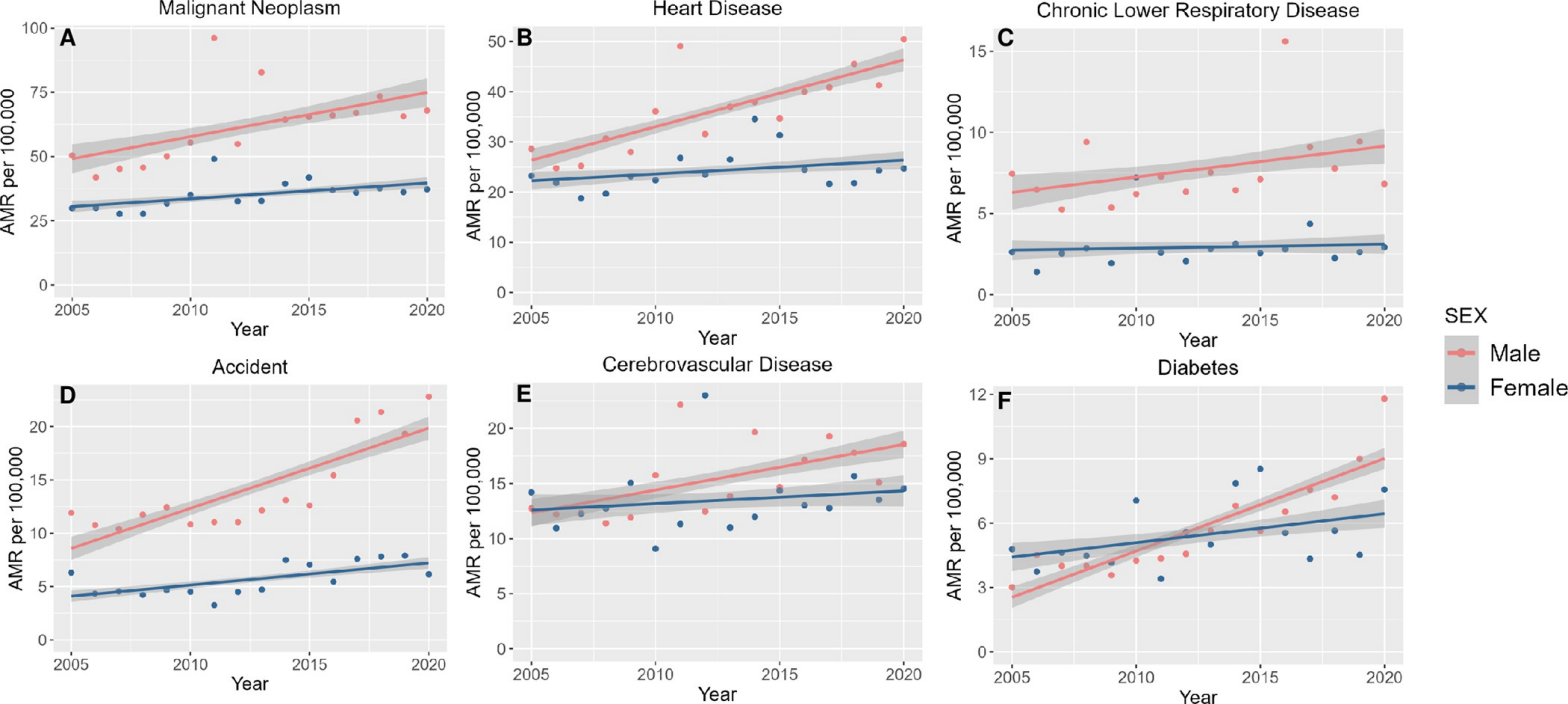
Age-standardized mortality rates from (A) cancer, (B) heart diseases, (C) chronic lower respiratory tract diseases, (D) accidents, (E) cerebrovascular diseases, and (F) diabetes among Vietnamese Americans stratified by sex, 2005–2020.

**Fig 3:**
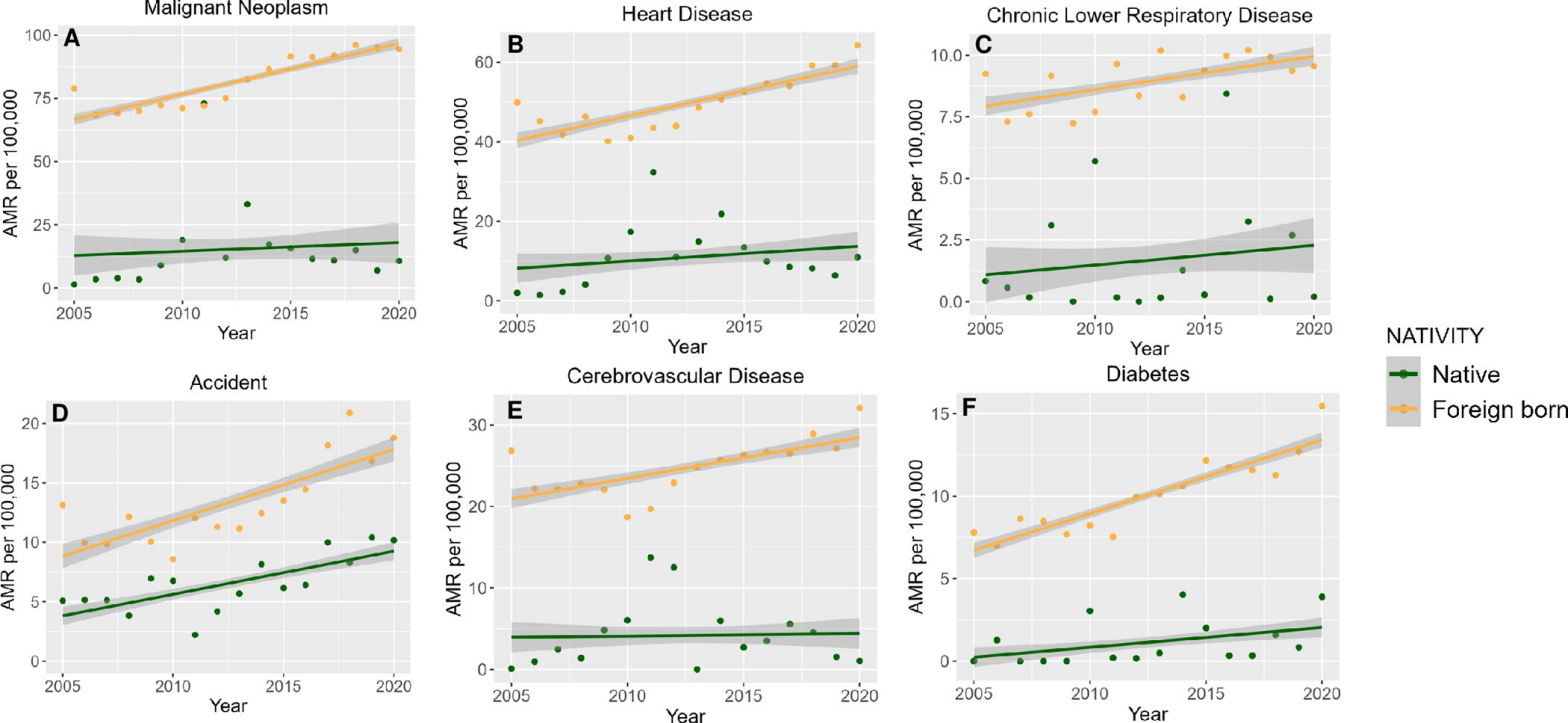
Age-standardized mortality rates from (A) cancer, (B) heart diseases, (C) chronic lower respiratory tract diseases, (D) accidents, (E) cerebrovascular diseases, and (F) diabetes among Vietnamese Americans stratified by nativity, 2005–2020.

When stratified by sex (**Fig 2**, corresponding data in **S2 Table**), VietAm male mortality increased from cancer, heart disease, chronic lower respiratory diseases, accidents, cerebrovascular disease, and diabetes (p<0.001 for all). By contrast, female VietAm mortality increased from cancer, heart disease, accidents, and diabetes (p<0.05), albeit at a slower rate than for males. For diabetes, VietAm male mortality was initially lower than VietAm female mortality (in year 2005); however, due to the more rapid increase in male mortality over the time period, by 2020 male mortality was higher. We also performed analysis stratified by nativity status (**Fig 3**, corresponding data in **S3 Table**). While differences in age structure between foreign- and native-groups make direct comparison challenging, certain observations can be made. Namely, foreign-born VietAms demonstrated increasing mortality from each of these leading causes over the study period (p<0.001 for all six causes). By contrast, native-born VietAms demonstrated increasing mortality only from accidents (APC 6.10%, p<0.001) and diabetes (APC 15.7%, p<0.001). We note that given the limited number of decedents within the native-born VietAm group, substantial year-to-year variability was seen.

## Discussion

In this study, we present the leading causes of mortality among VietAms stratified by age and nativity status; we also compare leading causes of mortality between VietAms, aggregated Asian Americans, and NHW Americans. We believe this is one of the first studies to incorporate national death data to understand patterns of mortality among the VietAm population. This study fills a critical gap in the existing literature, and highlights the importance of data disaggregation to allow for precision public health interventions, especially as the VietAm population is one of the largest and most rapidly growing Asian subgroups.

Our study offers several important observations. First, the ten leading causes of mortality accounted for ~75% of all deaths among VietAms, and ~73% of all deaths among NHWs. However among native-born VietAms, the comparable figure was only 54%. This is likely due to differences in the age structure between native-born and foreign-born populations, with native-born VietAms significantly younger. As the leading causes are mostly chronic and age-related diseases, their lower proportional mortality among the native-born is expected. For the same reason, the two leading causes of death (accidents 19%, self-harm 12%) among native-born VietAms are not a direct consequence of chronic disease. There exist notable differences in the leading causes of death between VietAms and Vietnamese nationals. The leading causes of death in Vietnam in 2017 are (in order) cerebrovascular disease (stroke), ischemic heart disease, cancer, and chronic respiratory disease [16]. It is interesting to note that among our VietAm cohort, heart disease surpassed stroke as a leading cause of death—the reverse of what is observed in Vietnam. This finding is not unique to Vietnamese. Stroke surpasses ischemic heart disease as a cause of death across the entirety of East Asia [17, 18]. Yet in the US, ischemic heart disease mortality exceeds stroke mortality for every Asian subgroup [19, 20]. It has been hypothesized that stroke risk is more strongly associated with hypertension and arteriosclerosis, whereas ischemic heart disease is associated with large-vessel atherosclerosis [17]. Therefore the inversion between stroke and ischemic heart disease risk seen in VietAms may warrant further public health attention and interventions focused on atherosclerotic risk factors.

When comparing VietAms with aggregated Asian Americans, it appears that the causes of mortality between VietAms and aggregated Asians are similar with regards to proportion, but lower on an absolute level in VietAms. Other studies have similarly suggested that VietAms may have lower rates of both cardiovascular [20] and overall mortality [19] compared to the broader Asian American population. Yet at the same time, secular trend analysis reveals these advantages to be rapidly eroding as VietAm mortality increased among all major causes. These trend differences become even more pronounced when comparing VietAms with NHWs. While NHWs had significantly higher mortality across disease classes compared to VietAms, NHW mortality decreased for nearly every leading cause over the study period. Our data supports the possibility of the “Healthy Immigrant Effect” described in the literature; immigrants have overall better health, but this advantage attenuates with increased length of residency [21–25]. Previous studies proposed that the selectivity of the immigration process explains the overall better health of immigrants. According to this theory, immigrants enjoy higher social status and baseline health compared to their peers (who lack the means or the skills to immigrate) [26, 27]. Our study cannot fully address the question of whether acculturation was a driver in the trend toward increased mortality over time, as we did not have access to individual-level data. However, the substantive trends toward increasing VietAm mortality observed in this study serve as an important warning on the need for future research focused on the vulnerable first-generation immigrant population.

Across age groups, male VietAms consistently demonstrated higher mortality compared to females. This is not a finding unique to the VietAm population, as males demonstrate higher mortality than females in every age group and in every region of the world [28]. This differential mortality remains even during times of extreme environmental stress, such as famines and epidemics [29]. This persistent observation is likely a complex interaction between genetic, biological, environmental, and social factors.

### Limitations

Our study has limitations. One significant limitation is the difference in age structure between native- and foreign-born populations. While we reported age-adjusted mortality rates, it may be that the age structure between the two groups are too different to fully control for in statistical analysis. As the native-born VietAm population ages into the 21^st^ century, it will be noteworthy to observe if this population follows the mortality patterns of their parental generation. There is the possibility of racial misclassification on death certificates. Prior reports have suggested that Asian individuals are sometimes reported as White on death certificates, thereby underestimating Asian mortality [30, 31]. It has been estimated that misclassification may cause an underestimation of the true mortality in Asians by 3–7% [31]. The comparable figure for disaggregated Asian subgroups is unknown but may be substantial. While the standard US death certificate began disaggregated Asian reporting in 2003, not all states adopted the new standard immediately [12]. As such, VietAm mortality may be underestimated in the early years of the period.

## Conclusion

VietAms are a distinct subgroup of Asian Americans with unique patterns of mortality. While VietAms have lower mortality compared to aggregated Asians and NHWs, these advantages are rapidly eroding over time. Future research needs to be focused on understanding and ultimately arresting the concerning trajectory of VietAm mortality across several disease classes.

## Supporting information

S1 Table Annual percent change (APC) of age-standardized mortality rates from cancer, heart diseases, chronic lower respiratory tract diseases, accidents, cerebrovascular diseases, and diabetes among Vietnamese Americans, aggregated Asian Americans and Non-Hispanic Whites. These values correspond to data from **Fig 1**.
(PDF)

S2 Table Annual percent change (APC) of age-standardized mortality rates from cancer, heart diseases, chronic lower respiratory tract diseases, accidents, cerebrovascular diseases, and diabetes among Vietnamese Americans by sex. These values correspond to data from **Fig 2**.
(PDF)

S3 Table Annual percent change (APC) of age-standardized mortality rates from cancer, heart diseases, chronic lower respiratory tract diseases, accidents, cerebrovascular diseases, and diabetes among Vietnamese Americans by nativity. These values correspond to data from **Fig 3.**
(PDF)

S1 Fig Flow chart summarizes the data collection, data engineering, and data analysis for analyzing the leading causes of death among Vietnamese Americans (VietAms), aggregated Asian Americans, and non-Hispanic Whites (NHWs). ACS, American Community Survey; NCHS, National Center for Health Statistics; UCOD, underlying cause of death.
(PDF)

S2 Fig Denominator population for non-US-born (foreign-born) and US-born (native-born) Vietnamese Americans in 2020. (PDF)

## Abbreviations

AMR: age-adjusted mortality rate per 100,000 individuals
APC: annual percent change
CI: confidence interval
ICD-10: International Classification of Diseases, 10^th^ revision
NHW: non-Hispanic White
US: United States
VietAm: Vietnamese American
