# Supplementary material for: Leading causes of death in Vietnamese Americans: An ecological study based on national death records from 2005–2020
Source: PLoS One. 2024 May 24;19(5):e0303195. doi: 10.1371/journal.pone.0303195 (PMC11125458; doi:10.1371/journal.pone.0303195)
Supplement: S1 Table — These values correspond to data from Fig 1. (PDF) [file pone.0303195.s001.pdf]

| S1 Table: Annual percent change of mortality by race, 2005-2020 | | | | | | |
|---|---|---|---|---|---|---|
| **Cause of death** | **Vietnamese American** | | **Aggregated Asian American** | | **Non-Hispanic White** | |
| | **APC** | **P-value** | **APC** | **P-value** | **APC** | **P-value** |
| Malignant Neoplasm | 2.46% | <0.001 | -1.11% | <0.001 | -1.43% | <0.001 |
| Heart Disease | 2.72% | <0.001 | -1.11% | <0.001 | -1.91% | <0.001 |
| Chronic Lower Respiratory Disease | 2.05% | 0.0031 | -1.1% | <0.001 | -0.91% | <0.001 |
| Accidents | 5.20% | <0.001 | 1.07% | <0.001 | 1.65% | <0.001 |
| Cerebrovascular Disease | 1.86% | <0.001 | 0.59% | 0.014 | -1.41% | <0.001 |
| Diabetes | 5.46% | <0.001 | 0.63% | <0.001 | -0.25% | 0.22 |

**S1 Table**: Annual percent change (APC) of age-standardized mortality rates from cancer, heart diseases, chronic lower respiratory tract diseases, accidents, cerebrovascular diseases, and diabetes among Vietnamese Americans, aggregated Asian Americans and Non-Hispanic Whites. These values correspond to data from **Figure 1**.
