# Supplementary material for: Leading causes of death in Vietnamese Americans: An ecological study based on national death records from 2005–2020
Source: PLoS One. 2024 May 24;19(5):e0303195. doi: 10.1371/journal.pone.0303195 (PMC11125458; doi:10.1371/journal.pone.0303195)
Supplement: S2 Table — These values correspond to data from Fig 2. (PDF) [file pone.0303195.s002.pdf]

S2 Table: Annual percent change of mortality among Vietnamese Americans by sex, 2005-2020				
**Cause of death**	**Male**		**Female**	
	**APC**	**P-value**	**APC**	**P-value**
Malignant Neoplasm	2.85%	<0.001	1.77%	<0.001
Heart Disease	3.84%	<0.001	1.13%	0.0097
Chronic Lower Respiratory Disease	2.52%	<0.001	0.81%	0.50
Accidents	5.76%	<0.001	3.85%	<0.001
Cerebrovascular Disease	2.76%	<0.001	0.87%	0.16
Diabetes	8.81%	<0.001	2.54%	<0.001

S2 Table: Annual percent change (APC) of age-standardized mortality rates from cancer, heart diseases, chronic lower respiratory tract diseases, accidents, cerebrovascular diseases, and diabetes among Vietnamese Americans by sex. These values correspond to data from **Figure 2**.
