# Supplementary material for: Leading causes of death in Vietnamese Americans: An ecological study based on national death records from 2005–2020
Source: PLoS One. 2024 May 24;19(5):e0303195. doi: 10.1371/journal.pone.0303195 (PMC11125458; doi:10.1371/journal.pone.0303195)
Supplement: S3 Table — These values correspond to data from Fig 3. (PDF) [file pone.0303195.s003.pdf]

S3 Table: Annual percent change of mortality among Vietnamese Americans by nativity, 2005-2020				
Cause of death	Native-born		Foreign-born	
	APC	P-value	APC	P-value
Malignant Neoplasm	2.25%	0.46	2.51%	<0.001
Heart Disease	3.50%	0.083	2.57%	<0.001
Chronic Lower Respiratory Disease	5.09%	0.22	1.53%	<0.001
Accidents	6.10%	<0.001	4.78%	<0.001
Cerebrovascular Disease	0.75%	0.77	2.07%	<0.001
Diabetes	15.68%	<0.001	4.72%	<0.001

S3 Table: Annual percent change (APC) of age-standardized mortality rates from cancer, heart diseases, chronic lower respiratory tract diseases, accidents, cerebrovascular diseases, and diabetes among Vietnamese Americans by nativity. These values correspond to data from **Figure 3**
