# Supplementary material for: Leading causes of death in Vietnamese Americans: An ecological study based on national death records from 2005–2020
Source: PLoS One. 2024 May 24;19(5):e0303195. doi: 10.1371/journal.pone.0303195 (PMC11125458; doi:10.1371/journal.pone.0303195)
Supplement: S1 Fig — ACS, American Community Survey; NCHS, National Center for Health Statistics; UCOD, underlying cause of death. (PDF) [file pone.0303195.s004.pdf]

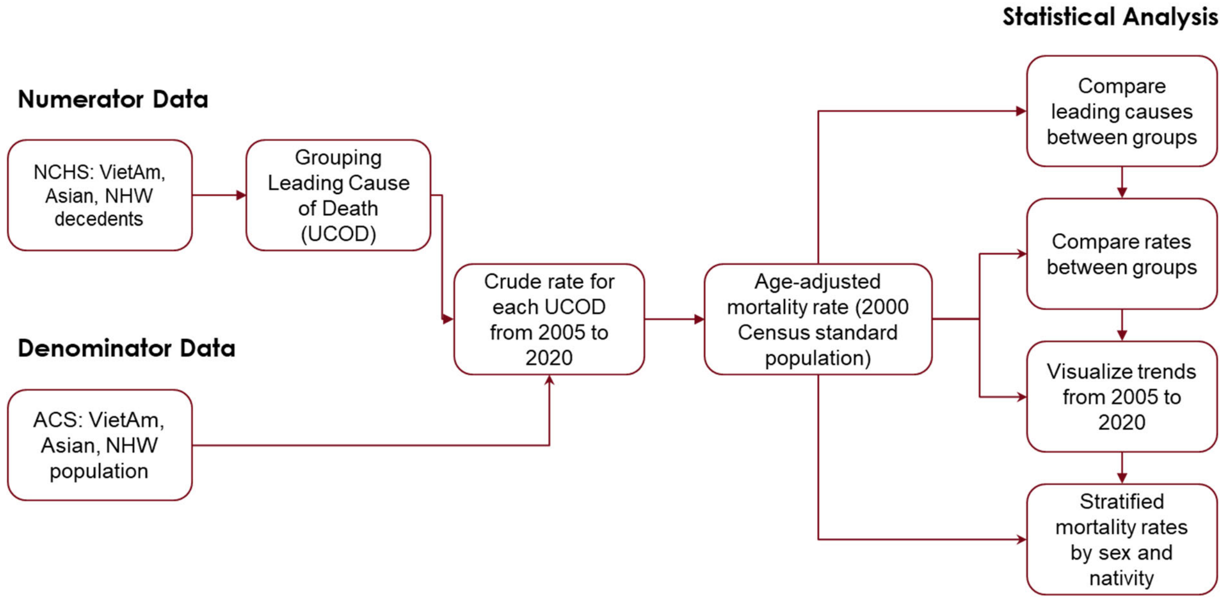


**S1 Figure:** Flow chart summarizes the data collection, data engineering, and data analysis for analyzing the leading causes of death among Vietnamese Americans (VietAms), aggregated Asian Americans, and non-Hispanic Whites (NHWs). ACS, American Community Survey; NCHS, National Center for Health Statistics; UCOD, underlying cause of death.
