# Supplementary material for: Leading causes of death in Vietnamese Americans: An ecological study based on national death records from 2005–2020
Source: PLoS One. 2024 May 24;19(5):e0303195. doi: 10.1371/journal.pone.0303195 (PMC11125458; doi:10.1371/journal.pone.0303195)
Supplement: S2 Fig — (PDF) [file pone.0303195.s005.pdf]

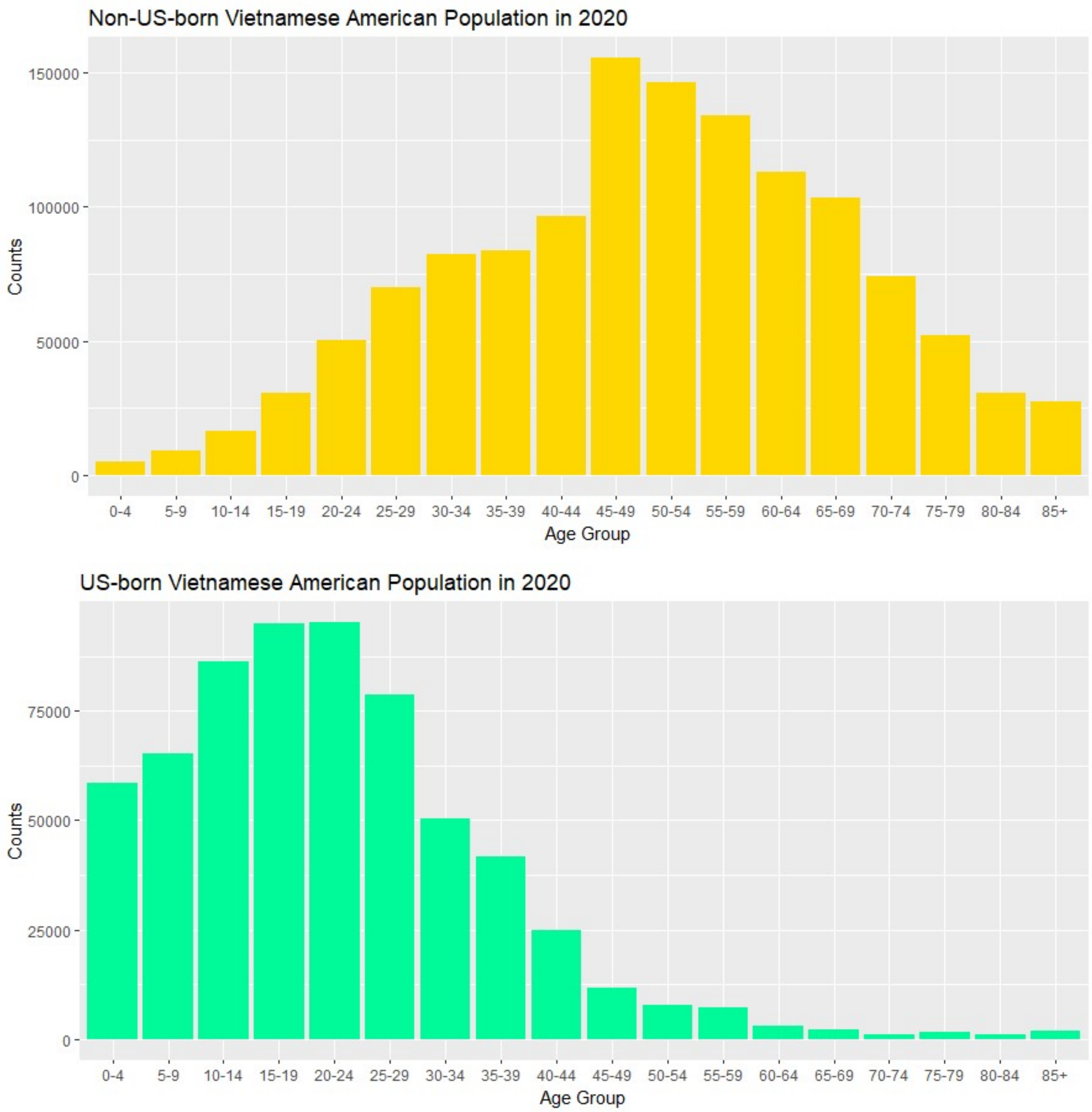

S2 Figure: Denominator population for non-US-born (foreign-born) and US-born (native-born) Vietnamese Americans in 2020.
